# The Transition of Social Isolation and Related Psychological Factors in 2 Mild Lockdown Periods During the COVID-19 Pandemic in Japan: Longitudinal Survey Study

**DOI:** 10.2196/32694

**Published:** 2022-03-08

**Authors:** Nagisa Sugaya, Tetsuya Yamamoto, Naho Suzuki, Chigusa Uchiumi

**Affiliations:** 1 Unit of Public Health and Preventive Medicine School of Medicine Yokohama City University Yokohama Japan; 2 Graduate School of Technology, Industrial and Social Sciences Tokushima University Tokushima Japan; 3 Graduate School of Sciences and Technology for Innovation Tokushima University Tokushima Japan

**Keywords:** coronavirus disease 2019, mild lockdown, social isolation, longitudinal survey, public health, surveillance, epidemiology, COVID-19, pandemic, lockdown, psychological behavior, social factors, mental health

## Abstract

**Background:**

Lockdowns and stay-at-home orders announced internationally for COVID-19 have led to physical and social distancing, with reports of many individuals experiencing social isolation (SI) and loneliness. Although the emergency declaration in Japan was declared as a “mild” lockdown requested by the government without penalties for violations, the lockdown measures, including SI, had several influences on people’s lives and mental health as in other countries. Furthermore, Japan declared a state of emergency multiple times; thus, it is necessary to examine the influence of the transition of SI caused by repeated emergency declarations and the deterioration of mental health associated with these changes.

**Objective:**

This study longitudinally investigated the transition of SI and its related factors during the mild lockdown under 2 declared states of emergency in Japan and analyzed psychosocial characteristics by extracting clusters where people with specific transition patterns of SI predominated.

**Methods:**

We collected data on 7893 inhabitants (3694 [46.8%] women, 49.6 [SD 13.7] years old) living in the 7 prefectures where the initial emergency declaration was applied. The investigations took place online in the final phase of the first and second states of emergency: phase 1 (between May 11 and 12, 2020) and phase 2 (between February 24 and 28, 2021). Nonparametric Bayesian coclustering was used to visualize the exhaustive interaction structure between the transition pattern of SI and the psychosocial variables.

**Results:**

There were no improvements in social networks and loneliness between the 2 phases, although psychological distress significantly improved and depression slightly decreased. Overall, 3868 (49%) of the 7893 participants remained socially isolated through phases 1 and 2, and 947 (12%) were socially isolated in phase 2, even though they were not socially isolated in phase 1. More participants experienced persistent SI in unmarried, childless, and low-household-income groups. The persistent-SI group had fewer cohabitants than other transition pattern groups. The nonparametric Bayesian coclustering results showed that most clusters, including participants without SI throughout phases 1 and 2, had healthy behaviors, more interactions, good relationships, and less loneliness and psychological stress. Furthermore, the cluster in which relationships deteriorated in phase 1 recovered in phase 2. Comparatively, the clusters with SI throughout phases 1 and 2 were divided into clusters with increased loneliness and psychological stress; clusters were close to participants’ average scores in this study. The clusters with increased loneliness and psychological stress were notable for deteriorating relationships and less online interaction.

**Conclusions:**

This study revealed the actual state of transition of SI and related psychological, social, and behavioral factors under repeated declarations of a state of emergency. These results should help construct intervention methods that fit individual characteristics of people in SI during a pandemic.

## Introduction

COVID-19 has rapidly spread worldwide since its outbreak in December 2019 [1]. To deter the spread of COVID-19, many countries have imposed a lockdown with restrictions on outings, service closures, etc. Although lockdowns are expected to prevent the spread of infection, they also cause psychological distress and economic damage [2-4].

Lockdowns and stay-at-home orders announced internationally for COVID-19 have led to physical and social distancing, with reports of many individuals experiencing social isolation (SI) [1,5,6]. Previous research in the elderly reported that individuals who were socially isolated before the pandemic were particularly vulnerable to the negative psychological impacts of the COVID-19 lockdown [6]. However, greater social support during the pandemic was reported to be inversely associated with thoughts of suicide and self-harm [7]. In addition, elevated loneliness during stay-at-home orders is strongly associated with more severe depression and suicidal ideation [8,9]. Thus, SI and the resulting loneliness under stay-at-home orders for COVID-19 are a critical public health concern.

The impact of the “mild” lockdown [10] following the declaration of a state of emergency in Japan has attracted attention. On April 7, 2020, the Japanese government declared a state of emergency due to the COVID-19 outbreak in 7 prefectures [11]. The state of emergency expanded nationwide on April 16, 2020, and was lifted in a phased manner starting on May 14, 2020. In the middle of the third wave of COVID-19, the Japanese government again declared a state of emergency in 4 prefectures on January 8, 2021, and 7 more on January 14. The second state of emergency was lifted in stages starting in March, except for 1 prefecture, where the state was lifted on February 7. Although many countries were in lockdown with penalties for violations, a distinguishing feature of the Japanese policy for COVID-19 was the government requesting that people refrain from going out, except for emergencies, and temporarily close certain businesses, with no penalties imposed for violations. As the emergency declaration in Japan was a “request” by the government, it did not prohibit people from going out or meeting other people. However, Japan’s mild lockdown influenced people’s lives in many ways, as in other countries, such as lifestyle changes due to teleworking or online classes held in many schools and economic damage due to decreased income or job loss.

Additionally, this lockdown significantly transformed activity in Japan; for example, the number of monthly train users in April 2020 and February 2021 prominently decreased by 45.5% compared to the previous year [12,13]. Our previous research reported severe SI, loneliness, and psychological distress during the first mild lockdown in Japan [5,10,14,15]. Furthermore, our previous study reported that people experienced extreme SI in the first state of emergency. Again, being male, being middle aged, and having a lower income predicted SI. In contrast, being a student was inversely associated with SI [5].

However, just as many countries have repeatedly declared lockdowns, Japan has also repeatedly declared emergencies, as mentioned earlier. Therefore, it is necessary to examine the influence of the transition (ie, worsening, improving, or maintaining) of SI caused by the prolonged pandemic and emergency declarations and the relationship between the transition pattern and the deterioration of mental health associated with the prolonged pandemic [16]. By examining these findings, we might clarify whether SI in the pandemic is a persistent problem rather than a temporary one and whether people who did not show SI problems in the early stages of the pandemic will later reveal such issues. In addition, clarifying the psychosocial characteristics of people who manifest different transition patterns of SI can provide information to consider what kind of help is needed based on individuals’ characteristics.

Therefore, the purpose of this study was to longitudinally investigate the transition of SI from the beginning of the pandemic to the end of a specified period, and its related factors by surveying during the mild lockdown under 2 declared states of emergency in Japan. We also analyzed psychosocial characteristics by extracting clusters where people with specific transition patterns of SI (development or maintenance of a state of SI in particular) predominated.

## Methods

### Participants and Data Collection

The survey was conducted online between May 11 and 12, 2020 (phase 1) and between February 24 and 28, 2021 (phase 2), the final phase of the state of emergency. In phase 1, we conducted an online survey of inhabitants living in the 7 prefectures where the emergency declaration measures were first applied (Tokyo, Kanagawa, Osaka, Saitama, Chiba, Hyogo, and Fukuoka) in order to detect precisely the impact of the mild lockdown. We conducted a follow-up survey on the same participants in phase 2. We recruited participants according to the following inclusion criteria: (1) inhabitants living in the 7 prefectures mentioned earlier and (2) age≥20 years. The exclusion criteria were as follows: (1) age<18 years, (2) high school students, and (3) living outside the seven prefectures. We determined that the target sample in phase 1 was 11,000 because of the possibility of dropouts from the follow-up survey and the large sample size required for nonparametric Bayesian coclustering with many variables. These prefectures were assumed to be susceptible to a mild lockdown due to their large populations and the large number of COVID-19 cases reported in these areas. In phase 1, the number of people in each prefecture was determined based on the ratio of the number of people living in Tokyo (n=2783, 24.6%), Kanagawa (n=1863, 16.4%), Osaka (n=1794, 15.8%), Saitama (n=1484, 13.1%), Chiba (n=1263, 11.1%), Hyogo (n=1119, 9.9%), and Fukuoka (n=1027, 9.1%).

The participants of this study were recruited through Macromill, Inc (Tokyo, Japan), a global marketing research company. This company has access to more than 1,300,000 registered members with diverse characteristics regarding sex and age of all prefectures in Japan. This online survey system automatically eliminated duplicate answers from a single respondent. Approximately 80,000 registered people who lived in the target areas were recruited by email, and data were collected on an online platform. (The target sample in phase 1 was n=11,000.) Participants completed the online survey after receiving a link to it. All participants voluntarily responded to the survey anonymously and provided informed consent online before completing the survey. Participants received a clear explanation of the survey procedure and could interrupt or terminate the survey at any time without requiring a reason. The questionnaire format, excluding the default items provided by Macromill, Inc (sex, age, occupation, annual household income, marital status, and presence of children) did not allow participants to proceed to the next page if there were items they had not answered. All the participants received Macromill points for their participation, which constitute an original point service of Macromill, Inc, and the participants can exchange these points for prizes or cash.

This study was approved by the Research Ethics Committee at the Graduate School of Social and Industrial Science and Technology, Tokushima University (acceptance no. 212). The study was performed in accordance with the ethical standards of the 1964 Declaration of Helsinki and its later amendments.

The data for this study were partly extracted from a database containing data used in our previous paper [16]. The extracted data were secondarily reanalyzed with different dependent and independent variables compared to those in the studies mentioned earlier.

### Measurements

#### Sociodemographic Data

We collected participants’ sociodemographic information, including age, sex, employment status (employed, homemaker, student, unemployed, or other), marital status, and annual household income (<JPY 2.0 million, JPY 2.0–3.9 million, JPY 4.0–5.9 million, JPY 6.0–7.9 million, ≥JPY 8.0 million, or unknown; a currency exchange rate of JPY 1=US $0.0086 is applicable). The details of the survey items are available on an open data platform (Open Science Framework). In addition, information was collected on whether the individual or a family member was a health care worker, was currently being treated for a mental condition or severe physical disease, and had a history of treatment for a mental disorder or severe physical illness. This information was used to compare the impact on the group assumed to be vulnerable to the lockdown effects in previous studies [17-20]. Although this information was collected in phase 1, the number of cohabitants was included in the survey in phase 2. Therefore, we needed to confirm whether the number of cohabitants could affect the SI scores of our participants, as the response option for the SI scale in this study was the number of people in their social network.

#### Social Isolation

Since the emergency declaration, we measured social networks using the Japanese version of the abbreviated Lubben Social Network Scale (LSNS-6, [21]). The LSNS-6 is a shortened version of the Lubben Social Network Scale [22] that includes items on the network size of relatives or friends who provide emotional and instrumental support. The LSNS-6 consists of 3 items related to the family network and 3 related to the friendship network.

The number of people in the network was calculated using a 6-point scale (0=none; 1=1 person; 2=2 people, 3=3 or 4 people; 4=5-8 people; and 5=9 or more people) for each item [23]. The total score ranged from 0 to 30 points, with higher scores indicating a larger social network and <12 points indicating SI. An LSNS-6 score of <12 points varied strongly related to sociodemographic and socioeconomic factors [24], while the score predicted depression and the development of poor physical capability [25]. The Cronbach α coefficient of the LSNS-6 for our data in phase 1 was .859.

#### Loneliness

We measured loneliness using the Japanese version of the University of California, Los Angeles (UCLA) Loneliness Scale, Version 3 (UCLA-LS3, [26]). The UCLA-LS3 consists of 10 items, each rated from 1 (never) to 4 (always) [27]. The total scores ranged from 10 to 40, with higher scores indicating higher levels of loneliness. The Cronbach α coefficient of the UCLA-LS3 for our data in phase 1 was .868. Loneliness and SI are conceptually distinct, with SI generally defined in terms of the objective availability of social contacts and the frequency of contact with social network members. In contrast, loneliness refers to the perception that personal and social needs are not being met [28,29]. Moreover, SI has been reported to relate to loneliness and is often a risk factor [30].

#### Psychological Distress

Psychological distress was measured using the Japanese version of the Kessler Psychological Distress Scale-6 (K6, [31]), a nonspecific psychological stress scale, and a 6-item screening instrument measuring distress over the past 30 days. Each question was rated on a scale of 0 (never) to 4 (always), with total scores ranging from 0 to 24. Owing to its brevity and high accuracy, the K6 is considered an ideal scale for screening for mental disorders in population-based health surveys as it is brief and highly accurate [31-33]. The Cronbach α coefficient of K6 for our data in phase 1 was .913.

We also used the Japanese version of the Patient Health Questionnaire-9 (PHQ-9, [34]) to collect basic information about the participants’ mental health; the PHQ-9 consists of 9 questions. Participants reported depressive symptoms during the past 4 weeks, with a score of 0 (none) to 3 (nearly every day) [35]. The Cronbach α coefficient of PHQ-9 for our data in phase 1 was .910.

#### Lifestyle, Coping Behavior, and Stressors Related to the Mild Lockdown

With extensive references to the literature on the COVID-19 pandemic [17,19,20,36,37], we developed 8 lifestyle and coping behavior items and 7 stressors were assumed to be associated with the mild lockdown (refer to [15,38] and Multimedia Appendix 1). We asked participants to rate the frequency of implementation and experience of these items from the start of the mild lockdown to the time of the survey on a scale of 1 (not at all) to 7 (extremely). Item details are described in our published papers [15,38]. This study treated these Likert scale values as interval scales for convenience, and parametric tests were performed on them.

### Statistical Analysis

The LSNS-6 scores of phases 1 and 2 were classified into 2 groups based on the cut-off point (12 points): with and without SI. The participants were further divided into the following 4 groups: those with no SI in both phases 1 and 2 (no-SI group), those with SI in phase 1 but not in phase 2 (improved-SI group), those with no SI in phase 1 but SI in phase 2 (worsened-SI group), and those with SI in phases 1 and 2 (persistent-SI group). The chi-square test and the *t* test were applied to compare sociodemographic characteristics and psychological indexes (LSNS-6, UCLA-LS3, K6, and PHQ-9) between individuals who participated only in phase 1 and individuals who participated in phases 1 and 2. The chi-square test compared sociodemographic data between the 4 groups. Additionally, repeated 2-way ANOVA was conducted to compare psychological indexes and mild-lockdown items for COVID-19 between the SI groups and between phases. Nonparametric Bayesian coclustering [39] visualized the exhaustive interaction structure between the transition pattern of SI and psychosocial variables exceeding the lower limit of the small effect size when comparing the 4 SI groups. These variables were not strongly correlated with others (ie, r<0.7). We selected the variable that had a more prominent group difference. Overall, 15,000 iterations based on the Bayesian optimization principle were performed to calculate the log marginal likelihood, which indicates the goodness of fit of the model. The log marginal probabilities were computed among the models, and the model with the highest log marginal likelihood was adopted. We converted the continuous variables to z values and assigned values between –3 and 3 to each isolation group according to the z value range: –3 for the no-SI group, –1 for the improved-SI group, 1 for the worsened-SI group, and 3 for the persistent-SI group. For all tests, significance was set at α=.05, 2-tailed. Statistical analyses were performed using SPSS Statistics version 25.0 (IBM Corp, NY, USA), MATLAB R2017a (Mathworks, Inc., Natick, MA, USA), and RStudio version 1.1.442.

## Results

### Descriptive Results

Table 1 shows the sociodemographic characteristics in our data. In phase 1, a total of 11,333 individuals participated, and we conducted a follow-up survey on them in phase 2. A total of 7893 individuals participated in phases 1 and 2 (3694 [46.8%] women, mean age 49.6 [SD 13.7] years, range 18-89 years), and thus, 3440 (30.35%) of 11,333 individuals who participated in phase 1 did not respond in phase 2. In addition, significantly more females than males participated only in phase 1. Individuals who participated only in phase 1 had significantly higher LSNS-6, K6, and PHQ-9 scores and substantially lower ages and UCLA-LS3 scores than individuals who participated both in phases 1 and 2 (Multimedia Appendix 2).

Regarding the number of people (N=7893) in each group classified based on the cut-off point of the LSNS-6, the no-SI group had 2296 (29.1%), the improved-SI group had 765 (9.7%), the worsened-SI group had 964 (12.2%), and the persistent-SI group had 3868 (49.0%) people (Table 1). The number of cohabitants in each SI group was 2.4 (SD1.4) in the no-SI group, 2.2 (SD 1.4) in the improved-SI group, 2.2 (SD 1.3) in the worsened-SI group, and 1.8 (SD 1.2) in the persistent-SI group. There was a significant difference in the number of cohabitants between SI groups (*F*_3_=106.79, *P*<.001, *η*^2^=0.039). Multiple comparisons showed that the persistent-SI group had significantly fewer cohabitants than other SI groups. The worsened-SI group had considerably fewer cohabitants than the no-SI group (all *P*<.001).

**Table 1:** Comparison of sociodemographic characteristics by transition pattern of SI^a^.

Sociodemographic indexes at time 1		Total, n (%)	LSNS^b^ group					Group difference				
			No SI, n (%)	Improved SI, n (%)	Worsened SI, n (%)	Persistent SI, n (%)	*χ*^2^ (*df*)		*P* value		Cramer *V*^c^	
Overall		7893 (100%)	2296 (29.1)	765 (9.7)	964 (12.2)	3868 (49.0)	N/A^d^		N/A		N/A	
**Sex**								71.62 (3)		<.001		0.095
	Male	4201 (53.2)	1061 (25.3) [–]^e^	427 (10.2)	503 (12.0)	2210 (52.6) [+]^f^	N/A		N/A		N/A	
	Female	3692 (46.8)	1235 (33.5) [+]	338 (9.2)	461 (12.5)	1658 (44.9) [–]	N/A		N/A		N/A	
**Age (years)**								99.92 (6)		<.001		0.080
	18-39	1926 (24.4)	620 (32.2) [+]	189 (9.8)	260 (13.5) [+]	857 (44.5) [–]	N/A		N/A		N/A	
	40-64	4714 (59.7)	1213 (25.7) [–]	437 (9.3)	563 (11.9)	2501 (53.1) [+]	N/A		N/A		N/A	
	≥65	1253 (15.9)	463 (37.0) [+]	139 (11.1)	141 (11.3)	510 (40.7) [–]	N/A		N/A		N/A	
**Occupation**								113.53 (12)		<.001		0.069
	Employed	5384 (68.2)	1501 (27.9) [–]	521 (9.7)	700 (13.0) [+]	2662 (49.4)	N/A		N/A		N/A	
	Homemaker	1236 (15.7)	470 (38.0) [+]	125 (10.1)	133 (10.8)	508 (41.1) [–]	N/A		N/A		N/A	
	Student	111 (1.4)	53 (47.7) [+]	11 (9.9)	14 (12.6)	33 (29.7) [–]	N/A		N/A		N/A	
	Unemployed	901 (11.4)	207 (23.0) [–]	83 (9.2)	90 (10.0) [–]	521 (57.8) [+]	N/A		N/A		N/A	
	Other	261 (3.3)	65 (24.9)	25 (9.6)	27 (10.3)	144 (55.2) [+]	N/A		N/A		N/A	
**Marital status**								241.29 (3)		<.001		0.175
	Married	5174 (65.6)	1727 (33.4) [+]	546 (10.6) [+]	689 (13.3) [+]	2212 (42.8) [–]	N/A		N/A		N/A	
	Unmarried	2719 (34.4)	569 (20.9) [–]	219 (8.1) [–]	275 (10.1) [–]	1656 (60.9) [+]	N/A		N/A		N/A	
**Children**								313.05 (3)		<.001		0.199
	Yes	4477 (56.7)	1575 (35.2) [+]	490 (10.9) [+]	599 (13.4) [+]	1813 (40.5) [–]	N/A		N/A		N/A	
	No	3416 (43.3)	721 (21.1) [–]	275 (8.1) [–]	365 (10.7) [–]	2055 (60.2) [+]	N/A		N/A		N/A	
**Annual household income (JPY)^g^**								262.84 (12)		<.001		0.120
	<2.0 million	438 (5.5)	57 (13.0) [–]	23 (5.3) [–]	30 (6.8) [–]	328 (74.9) [+]	N/A		N/A		N/A	
	2.0-3.9 million	1421 (18.0)	319 (22.4) [–]	127 (8.9)	166 (11.7)	809 (56.9) [+]	N/A		N/A		N/A	
	4.0-5.9 million	1562 (19.8)	431 (27.6)	146 (9.3)	198 (12.7)	787 (50.4)	N/A		N/A		N/A	
	6.0-7.9 million	1078 (13.7)	326 (30.2)	118 (10.9)	145 (13.5)	489 (45.4) [–]	N/A		N/A		N/A	
	≥8.0 million	1613 (20.4)	627 (38.9) [+]	177 (11.0) [+]	196 (12.2)	613 (38.0) [–]	N/A		N/A		N/A	
**Health care worker (self)**								7.58 (3)		.06		0.031
	Yes	407 (5.2)	133 (32.7)	40 (9.8)	60 (14.7)	174 (42.8) [–]	N/A		N/A		N/A	
	No	7486 (94.8)	2163 (28.9)	725 (9.7)	904 (12.1)	3694 (49.3) [+]	N/A		N/A		N/A	
**Health care worker (family)**								38.26 (3)		<.001		0.070
	Yes	625 (7.9)	243 (38.9) [+]	61 (9.8)	81 (13.0)	240 (38.4) [–]	N/A		N/A		N/A	
	No	7268 (92.1)	2053 (28.2) [–]	704 (9.7)	883 (12.1)	3628 (49.9) [+]	N/A		N/A		N/A	
**Treatment of current severe physical diseases**								3.93 (3)		.27		0.022
	Yes	378 (4.8)	95 (25.1)	39 (10.3)	43 (11.4)	201 (53.2)	N/A		N/A		N/A	
	No	7515 (95.2)	2201 (29.3)	726 (9.7)	921 (12.3)	3667 (48.8)	N/A		N/A		N/A	
**Treatment of previous severe physical diseases** ** **								1.32 (3)		.72		0.013
	Yes	659 (8.3)	183 (27.8)	62 (9.4)	77 (11.7)	337 (51.1)	N/A		N/A		N/A	
	No	7234 (91.7)	2113 (29.2)	703 (9.7)	887 (12.3)	3531 (48.8)	N/A		N/A		N/A	
**Treatment of current psychological problems**								54.65 (3)		<.001		0.083
	Yes	431 (5.5)	63 (14.6) [–]	41 (9.5)	50 (11.6)	277 (64.3) [+]	N/A		N/A		N/A	
	No	7462 (94.5)	2233 (29.9) [+]	724 (9.7)	914 (12.2)	3591 (48.1) [–]	N/A		N/A		N/A	
**Treatment of previous psychological problems**								61.71 (3)		<.001		0.088
	Yes	875 (11.1)	167 (19.1) [–]	89 (10.2)	92 (10.5)	527 (60.2) [+]	N/A		N/A		N/A	
	No	7018 (88.9)	2129 (30.3) [+]	676 (9.6)	872 (12.4)	3341 (47.6) [–]	N/A		N/A		N/A	

^a^SI: social isolation.

^b^LSNS: Lubben Social Network Scale.

^c^Cramer *V*: 0.100, small; 0.300, medium; 0.600, large.

^d^N/A: not applicable.

^e^[–]: adjusted residuals≤–1.96.

^f^[+]: adjusted residuals≥1.96.

^g^In our data set, although 982 (12.4%) of 7893 participants did not provide any data regarding annual household income, there were no missing data for the other variables. The table does not include the “Unknown” classification of yearly household income (799/7893, 10.1%).

### Transition of Social Isolation and Sociodemographic Characteristics

Table 1 shows the differences in sociodemographic characteristics based on the transition pattern of SI. There were significant differences between the 4 groups of SI in all sociodemographic characteristics except for “Health worker (self)” and “Treatment of current/previous severe physical diseases” (ie, *P*<.05). Results of the chi-square test that exceeded the lower limit of “small effect size” (ie, Cramer *V*>0.100) indicated unmarried or childless people were more prevalent in the persistent-SI group. In contrast, married people and individuals with children were more commonplace in other SI groups. Additionally, individuals in lower-annual-household-income groups (<JPY 200 million or between JPY 2.0 and 3.9 million) were more prevalent in the persistent-SI group. Individuals in higher-annual-household-income groups (≥JPY 8.0 million) were more prevalent in the no-SI and improved-SI groups.

### Transition of Social Isolation and Psychological or COVID-19 Related Variables

Tables 2-4 display the differences and interactions between phases and the transition of SI in psychological or COVID-19-related variables. Regarding interactions between phases and groups, the results were significant on the LSNS-6, UCLA-LS3, K6, and items about lifestyle and stress management during the mild lockdown (“Deterioration of relationship with familiar people,” “Difficulties owing to the lack of daily necessities,” and “Difficulties in work or schoolwork”). In contrast, the results only on the LSNS-6 exceeded the lower limit of “small effect size” (ie, generalized *η*^2^ [*η*_G_^2^]>0.010). The simple main effect test in the LSNS-6 indicated a significant difference between each group in phases 1 and 2 and between phases in all groups.

Group classification had significant effects on all variables except COVID-19-related anxiety. At the same time, the results exceeded the lower limit of “small effect size” on the LSNS-6, UCLA-LS3, K6, PHQ-9, and lifestyle and stress management items during the mild lockdown and the “Deterioration of relationship with familiar people” item. The multiple comparison test indicated significant differences between all groups, excluding the improved-SI group and the worsened-SI group, on the LSNS-6, UCLA-LS3, K6, PHQ-9, and lifestyle and stress management items during the mild lockdown. Regarding “Deterioration of relationships with familiar people,” a significant difference between the no-SI group and other groups was evident.

Regarding the effect of phase, results were significant for all variables except for “Healthy sleep habits.” In contrast, results exceeded the lower limit of “small effect size” on the K6 as well as items on “Online interaction with familiar people,” “COVID-19-related anxiety,” “Difficulties owing to the lack of daily necessities,” and “Difficulties in work and schoolwork.”

**Table 2:** Differences and interactions between phases^a^ and transition of SI^b^ on different scales.

	Phase	Mean score (SD)					Effect of phase				Effect of group				Interaction		
		No SI	Improved SI	Worsened SI	Persistent SI	*F* (*df*)		*P* value	*η_G^2^_^c^*	*F* (*df*)		*P* value	*η_G^2^_*	*F* (*df*)		*P* value	*η_G^2^_*
**LSNS-6^d^**																	
	1	16.84 (3.71)	7.89 (2.77)	14.58 (2.73)	5.50 (3.31)	15.18 (1, 7889)		<.001	0.000	8071.80 (3, 7889)		<.001	0.640	2046.36 (3, 7889)		<.001	0.066
	2	16.46 (3.55)	14.56 (2.83)	7.81 (2.93)	5.21 (3.28)	N/A^e^		N/A	N/A	N/A		N/A	N/A	N/A		N/A	N/A
**UCLA-LS3^f^**																	
	1	19.56 (4.67)	23.07 (4.61)	22.26 (4.55)	26.41 (5.20)	10.51 (1, 7889)		.001	0.000	1096.28 (3, 7889)		<.001	0.259	38.59 (3, 7889)		<.001	0.002
	2	19.87 (4.86)	22.13 (4.76)	23.38 (4.59)	26.62 (5.33)	N/A		N/A	N/A	N/A		N/A	N/A	N/A		N/A	N/A
**K6^g^**																	
	1	3.99 (4.38)	5.23 (5.37)	4.96 (4.88)	6.12 (5.77)	536.70 (1, 7889)		<.001	0.013	103.10 (3, 7889)		<.001	0.030	2.64 (3, 7889)		.048	0.000
	2	2.63 (4.01)	3.37 (4.87)	3.68 (4.82)	4.71 (5.63)	N/A		N/A	N/A	N/A		N/A	N/A	N/A		N/A	N/A
**PHQ-9^h^**																	
	1	3.05 (4.06)	4.41 (5.39)	4.19 (5.05)	5.53 (5.93)	168.90 (1, 7889)		<.001	0.004	126.46 (3, 7889)		<.001	0.038	2.32 (3, 7889)		.073	0.000
	2	2.4 (3.89)	3.27 (5.19)	3.49 (4.88)	4.77 (6.07)	N/A		N/A	N/A	N/A		N/A	N/A	N/A		N/A	N/A

^a^Phase 1: between May 11 and 12, 2020, in the final phase of the first state of emergency; phase 2: between February 24 and 28, 2021, in the final phase of the second state of emergency.

^b^SI: social isolation.

^c^*η*_G_^2^: 0.010, small; 0.060, medium; 0.140, large.

^d^LSNS-6: Lubben Social Network Scale (shortened version).

^e^N/A: not applicable.

^f^UCLA-LS3: University of California, Los Angeles (UCLA) Loneliness Scale, Version 3.

^g^K6: Kessler Psychological Distress Scale-6.

^h^PHQ-9: Patient Health Questionnaire-9.

**Table 3:** Differences and interactions between phases^a^ and transition of SI^b^ with regard to lifestyle and coping behavior during the mild lockdown.

	Phase	Mean score (SD)					Effect of phase				Effect of group				Interaction		
		No SI	Improved SI	Worsened SI	Persistent SI	*F* (*df*)		*P* value	*η*_G^2^_^c^	*F* (*df*)		*P* value	*η*_G^2^_	*F* (*df*)		*P* value	*η*_G^2^_
**Exercise**																	
	1	4.70 (1.64)	4.19 (1.74)	4.29 (1.73)	3.73 (1.84)	231.42 (1, 7889)		<.001	0.007	172.62 (3, 7889)		<.001	0.047	9.25 (3, 7889)		<.001	0.001
	2	4.27 (1.85)	4.04 (1.80)	3.69 (1.87)	3.37 (1.92)	N/A^d^		N/A	N/A	N/A		N/A	N/A	N/A		N/A	N/A
**Healthy eating habits**																	
	1	4.85 (1.37)	4.29 (1.50)	4.50 (1.39)	4.02 (1.58)	59.83 (1, 7889)		<.001	0.002	174.34 (3, 7889)		<.001	0.049	11.40 (3, 7889)		<.001	0.001
	2	4.64 (1.48)	4.38 (1.62)	4.18 (1.58)	3.84 (1.67)	N/A		N/A	N/A	N/A		N/A	N/A	N/A		N/A	N/A
**Healthy sleep habits**																	
	1	5.11 (1.61)	4.57 (1.74)	4.93 (1.62)	4.48 (1.80)	0.36 (1, 7889)		.55	0.000	95.49 (3, 7889)		<.001	0.025	9.59 (3, 7889)		<.001	0.001
	2	5.15 (1.62)	4.81 (1.65)	4.71 (1.73)	4.48 (1.83)	N/A		N/A	N/A	N/A		N/A	N/A	N/A		N/A	N/A
**Favorite activity**																	
	1	4.47 (1.51)	3.96 (1.63)	4.18 (1.53)	3.68 (1.68)	165.71 (1, 7889)		<.001	0.006	140.84 (3, 7889)		<.001	0.037	14.76 (3, 7889)		<.001	0.002
	2	4.09 (1.64)	3.95 (1.65)	3.64 (1.64)	3.38 (1.70)	N/A		N/A	N/A	N/A		N/A	N/A	N/A		N/A	N/A
**Offline interaction with familiar people**																	
	1	4.10 (1.82)	3.54 (1.76)	3.82 (1.77)	3.12 (1.78)	80.24 (1, 7889)		<.001	0.003	248.96 (3, 7889)		<.001	0.057	8.41 (3, 7889)		<.001	0.001
	2	3.85 (1.77)	3.54 (1.74)	3.30 (1.72)	2.84 (1.73)	N/A		N/A	N/A	N/A		N/A	N/A	N/A		N/A	N/A
**Online interaction with familiar people**																	
	1	3.82 (2.01)	3.15 (1.85)	3.53 (1.92)	2.54 (1.73)	383.57 (1, 7889)		<.001	0.015	294.38 (3, 7889)		<.001	0.070	25.13 (3, 7889)		<.001	0.003
	2	3.10 (1.89)	2.84 (1.76)	2.72 (1.77)	2.18 (1.56)	N/A		N/A	N/A	N/A		N/A	N/A	N/A		N/A	N/A
**Preventive behaviors of COVID-19**																	
	1	5.92 (1.38)	5.41 (1.68)	5.65 (1.47)	5.25 (1.82)	8.39 (1, 7889)		.004	0.000	93.90 (3, 7889)		<.001	0.024	14.08 (3, 7889)		<.001	0.002
	2	5.83 (1.39)	5.51 (1.62)	5.30 (1.74)	5.29 (1.79)	N/A		N/A	N/A	N/A		N/A	N/A	N/A		N/A	N/A
**Optimism**																	
	1	4.65 (1.34)	3.94 (1.47)	4.25 (1.42)	3.57 (1.53)	65.02 (1, 7889)		<.001	0.002	336.67 (3, 7889)		<.001	0.081	18.09 (3, 7889)		<.001	0.002
	2	4.76 (1.41)	4.41 (1.51)	4.18 (1.46)	3.79 (1.55)	N/A		N/A	N/A	N/A		N/A	N/A	N/A		N/A	N/A

^a^Phase 1: between May 11 and 12, 2020, in the final phase of the first state of emergency; phase 2: between February 24 and 28, 2021, in the final phase of the second state of emergency.

^b^SI: social isolation.

^c^*η*_G_^2^: 0.010, small; 0.060, medium; 0.140, large.

^d^N/A: not applicable.

**Table 4:** Differences and interactions between phases^a^ and transition of SI^b^ with regard to stressors related to the mild lockdown.

	Phase	Mean score (SD)					Effect of phase				Effect of group				Interaction		
		No SI	Improved SI	Worsened SI	Persistent SI	*F* (*df*)		*P* value	*η*_G^2^_^c^	*F* (*df*)		*P* value	*η*_G^2^_	*F* (*df*)		*P* value	*η*_G^2^_
**Deterioration of household economy**																	
	1	3.55 (1.77)	3.70 (1.71)	3.78 (1.77)	3.81 (1.77)	119.88 (1, 7889)		<.001	0.004	16.53 (3, 7889)		<.001	0.005	0.31 (3, 7889)		.82	0.000
	2	3.27 (1.73)	3.41 (1.65)	3.49 (1.69)	3.56 (1.76)	N/A^d^		N/A	N/A	N/A		N/A	N/A	N/A		N/A	N/A
**Deterioration of relationship with familiar people**																	
	1	2.09 (1.42)	2.51 (1.53)	2.33 (1.44)	2.53 (1.55)	63.22 (1, 7889)		<.001	0.003	41.39 (3, 7889)		<.001	0.011	8.68 (3, 7889)		<.001	0.001
	2	2.34 (1.53)	2.60 (1.52)	2.67 (1.55)	2.61 (1.57)	N/A		N/A	N/A	N/A		N/A	N/A	N/A		N/A	N/A
**Frustration**																	
	1	3.04 (1.69)	3.27 (1.73)	3.27 (1.66)	3.35 (1.76)	7.52 (1, 7889)		.01	0.000	20.07 (3, 7889)		<.001	0.006	2.56 (3, 7889)		.05	0.000
	2	2.98 (1.70)	3.11 (1.67)	3.32 (1.72)	3.24 (1.74)	N/A		N/A	N/A	N/A		N/A	N/A	N/A		N/A	N/A
**COVID-19-related anxiety**																	
	1	4.04 (1.66)	3.97 (1.66)	4.07 (1.64)	3.98 (1.71)	418.68 (1, 7889)		<.001	0.015	0.65 (3, 7889)		.58	0.000	1.46 (3, 7889)		.22	0.000
	2	3.48 (1.68)	3.54 (1.63)	3.54 (1.65)	3.49 (1.71)	N/A		N/A	N/A	N/A		N/A	N/A	N/A		N/A	N/A
**COVID-19-related sleeplessness**																	
	1	2.33 (1.49)	2.55 (1.54)	2.57 (1.56)	2.50 (1.52)	4.57 (1, 7889)		.03	0.000	10.07 (3, 7889)		<.001	0.003	0.95 (3, 7889)		.42	0.000
	2	2.31 (1.46)	2.52 (1.56)	2.51 (1.51)	2.42 (1.48)	N/A		N/A	N/A	N/A		N/A	N/A	N/A		N/A	N/A
**Difficulties owing to the lack of daily necessities**																	
	1	3.45 (1.79)	3.48 (1.79)	3.61 (1.84)	3.65 (1.84)	1037.98 (1, 7889)		<.001	0.046	5.91 (3, 7889)		<.001	0.001	4.41 (3, 7889)		.004	0.000
	2	2.53 (1.59)	2.70 (1.62)	2.69 (1.57)	2.61 (1.58)	N/A		N/A	N/A	N/A		N/A	N/A	N/A		N/A	N/A
**Difficulties in work or schoolwork**																	
	1	3.64 (2.01)	3.65 (1.94)	3.79 (1.95)	3.56 (1.97)	746.47 (1, 7889)		<.001	0.028	4.43 (3, 7889)		.004	0.001	2.93 (3, 7889)		.03	0.000
	2	2.77 (1.74)	2.97 (1.78)	2.97 (1.75)	2.83 (1.75)	N/A		N/A	N/A	N/A		N/A	N/A	N/A		N/A	N/A

^a^Phase 1: between May 11 and 12, 2020, in the final phase of the first state of emergency; phase 2: between February 24 and 28, 2021, in the final phase of the second state of emergency.

^b^SI: social isolation.

^c^*η*_G_^2^: 0.010, small; 0.060, medium; 0.140, large.

^d^N/A: not applicable.

### Comprehensive Interaction Structure between Transition Pattern of Social Isolation and the Psychosocial Variables

**Figure 1:**
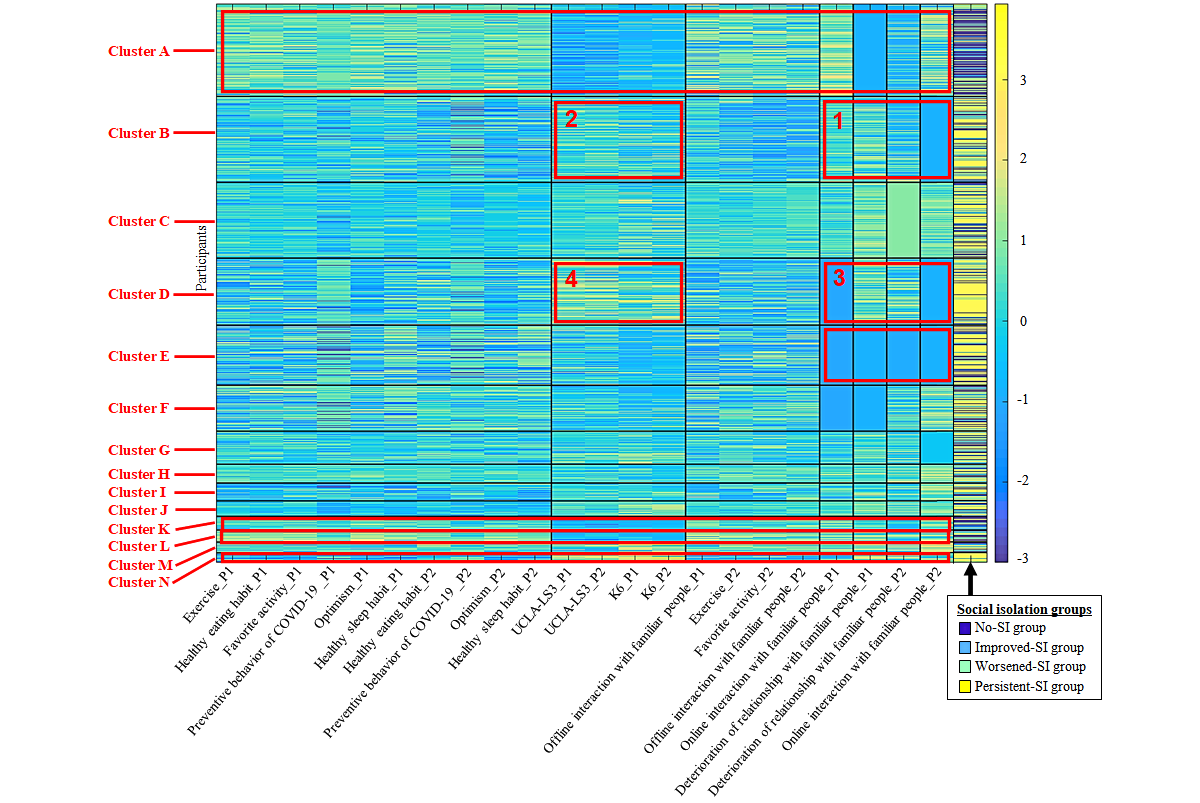
Comprehensive interaction structure of psychosocial variables associated with transition pattern of SI. The red boxes indicate variables with notable features in each cluster. K6: Kessler Psychological Distress Scale-6; P1: phase 1; P2: phase 2; SI: social isolation; UCLA-LS3: University of California, Los Angeles (UCLA) Loneliness Scale, Version 3.

The results of nonparametric Bayesian coclustering are shown in Figure 1 and Table 5. Clusters with more than 50% of the data in a particular group and adjusted residuals greater than 1.96 are clusters A, B, D, E, G, K, L, and N (Table 5, see values in italics). Of these clusters, we describe next clusters in which each variable had a notable feature (refer to red boxes in Figure 1). Multimedia Appendix 3 is a supplementary document displaying a visualization of differences in scores among the clusters.

For cluster A (720/1309 [55.0%] no-SI group in Table 5), healthy behaviors and attitudes, more online and offline interactions, decreased deterioration of relationships (especially in phase 1), and lower UCLA-LS3 and K-6 scores were maintained throughout phases 1 and 2 (see red box in Figure 1, cluster A).

For cluster B (661/1215 [54.4%] persistent-SI group in Table 5), the deterioration of relationships in phase 1 improved in phase 2. In contrast, online interactions, common in phase 1, decreased in phase 2 (see red box 1 in Figure 1, cluster B). The high UCLA-LS3 and K-6 scores were not higher than the average score throughout phases 1 and 2 (see red box 2 in Figure 1, cluster B).

Cluster D (725/947 [76.6%] persistent-SI group in Table 5) experienced deterioration of relationships, low online interactions (see red box 3 in Figure 1, cluster D), and high UCLA-LS3 and K-6 scores throughout phases 1 and 2 (see red box 4 in Figure 1, cluster D).

Cluster E (534/847 [63.0%] persistent-SI group in Table 5) experienced less deterioration of relationships and fewer online interactions throughout phases 1 and 2. Their scores on the UCLA-LS3 and K-6 were close to the mean scores of all participants (see red box in Figure 1, cluster E).

Cluster K (106/193 [54.9%] no-SI group in Table 5) maintained lower UCLA-LS3 and K-6 scores and high levels of online and offline interactions throughout phases 1 and 2 (see red box in Figure 1, bluster K).

Cluster L (100/171 [58.5%] no-SI group in Table 5) showed healthy behaviors and attitudes and high levels of online and offline interactions throughout phases 1 and 2. These characteristics were prominent, especially in cluster L. However, they maintained lower UCLA-LS3 and K-6 scores and deteriorated relationships, common in phase 1, and improved in phase 2 (see red box in Figure 1, cluster L).

Cluster N (110/138 [79.7%] persistent-SI group in Table 5) maintained fewer healthy behaviors and attitudes, fewer online and offline interactions, deterioration of relationships, and higher UCLA-LS3 and K-6 scores throughout phases 1 and 2 (see red box in Figure 1, cluster N).

In addition, we did not extract clusters for many improved-SI or worsened-SI groups.

**Table 5:** Number and percentage of each cluster in each SI^a^ group.

Results^b^			Cluster																							
		A		B		C	D		E		F		G		H		I	J		K		L		M		N
Total, N			1309		1215	1070		947		847		650		466		266	251		219		193		171		151	138
**No SI**																										
	n (%)	720 (*55.0*)		282 (23.2)		272 (25.4)	96 (10.1)		175 (20.7)		192 (29.5)		93 (20.0)		96 (36.1)		61 (24.3)	46 (21.0)		106 (*54.9*)		100 (*58.5*)		54 (35.8)		3 (2.2)
	Adjusted residuals	*22.6*		–4.9		–2.8	–13.7		–5.7		0.3		–4.5		2.6		–1.7	–2.7		*8.0*		*8.6*		1.8		–7.0
**Improved SI**																										
	n (%)	118 (9.0)		108 (8.9)		124 (11.6)	67 (7.1)		62 (7.3)		61 (9.4)		47 (10.1)		37 (13.9)		28 (11.2)	32 (14.6)		20 (10.4)		19 (11.1)		26 (17.2)		16 (11.6)
	Adjusted residuals	–0.9		–1.0		2.3	–2.9		–2.5		–0.3		0.3		2.4		0.8	2.5		0.3		0.6		3.2		0.8
**Worsened SI**																										
	n (%)	195 (14.9)		164 (13.5)		138 (12.9)	59 (6.2)		76 (9.0)		74 (11.4)		76 (16.3)		31 (11.7)		35 (13.9)	26 (11.9)		28 (14.5)		29 (17.0)		24 (15.9)		9 (6.5)
	Adjusted residuals	3.2		1.5		0.7	–6.0		–3.0		–0.7		2.8		–0.3		0.9	–0.2		1.0		1.9		1.4		–2.1
**Persistent SI**																										
	n (%)	276 (21.1)		661 (*54.4*)		536 (50.1)	725 (*76.6*)		534 (*63.0*)		323 (49.7)		250 (*53.6*)		102 (38.3)		127 (50.6)	115 (52.5)		39 (20.2)		23 (13.5)		47 (31.1)		110 (*79.7*)
	Adjusted residuals	–22.1		*4.1*		0.8	*18.1*		*8.7*		0.4		*2.1*		–3.5		0.5	1.1		–8.1		–9.4		–4.4		*7.3*

^a^SI: social isolation.

^b^Clusters A, B, D, E, G, K, L, and N had >50% of data in a particular group and adjusted residuals >1.96 (italicized values).

## Discussion

### Principal Findings

There were no improvements in social networks (the LSNS-6 score) and loneliness between the 2 emergency declarations, although psychological distress significantly improved and depression slightly decreased. This result may be because the same number of people in the group who had no SI problems (LSNS-6 score≤12) in phase 1 but became socially isolated (LSNS-6 score<12) in phase 2 and vice versa were included in the target population of this study. These groups were smaller than the group whose SI status remained unchanged. However, we confirmed that the LSNS-6 score and the UCLA-LS3 score observed at each time point tended to be lower and higher, respectively, than in previous studies conducted during the nonpandemic period; the mean score of the LSNS-6 was 16.2 points and that of UCLA-LS3 was 17.5 points [21,26]. Regarding the presence of SI in the 2 periods under the declaration of the state of emergency, 3868 (49%) of the 7893 participants remained socially isolated through both periods, and 947 (12%) were socially isolated at the time of the second declaration, even though they were not socially isolated at the time of the first declaration. Approximately 4815 (61%) of the 7893 participants became socially isolated at the time of the second declaration. At the same time, as a change in the psychological scale described earlier, there were significant decreases in online interactions with familiar people, COVID-19-related anxiety, difficulties owing to the lack of daily necessities, and difficulties in work or schoolwork. Although various COVID-19-related life problems improved and online connections decreased, which may correspond to the lack of improvement only in SI and loneliness. The comparisons by transition patterns of SI in these variables showed that individuals with persistent SI had severe loneliness, psychological distress, and depression; a worsened lifestyle and stress management during the mild lockdown; and deteriorated relationships with familiar people. Still, there were no significant differences between the improved- and worsened-SI groups. Additionally, there were no prominent interactions between transition patterns of SI and phases. Although various indicators changed across the participants, and various problems related to persistent SI were observed, no characteristics of time series changes in psychosocial variables in a specific transition pattern of SI were found.

Comparing the demographic data by transition patterns of SI, there were more unmarried or people without children with persistent SI. The persistent-SI group had fewer cohabitants than other transition pattern groups. This result is not surprising, since marital status and family structure are difficult to change. However, this result indicates that the number of people in the persistent-SI group is low, but also that there are few people with whom they can talk about their problems or whom they can ask for help. Therefore, this suggests that the mental health of people with these demographic characteristics should be of concern. In addition, more people in the low-income group had persistent SI, while more people in the high-income group had no SI or improved SI. At the time of the first emergency declaration [15], our previous results reported that low household income is associated with SI. Furthermore, this study indicated that among individuals who were socially isolated during the first emergency declaration, those with high incomes experienced less SI during the second declaration. However, this was not seen in many people with low incomes.

In the nonparametric Bayesian coclustering, the primary focus concerned clusters in which a particular transition pattern of SI predominated. Most of the clusters with participants without SI throughout phases 1 and 2 had healthy behaviors, more interactions, good relationships, and lower levels of loneliness and psychological stress. Furthermore, the clusters in which relationships deteriorated in phase 1 recovered in phase 2. Comparatively, the clusters with SI throughout phases 1 and 2 were further divided into clusters with increased loneliness and psychological stress and clusters close to the participants’ average scores in this study. Among these clusters, clusters with increased loneliness and psychological stress were notable for deteriorating relationships and fewer online interactions. These results suggest that even if the transition pattern of SI is similar, mental health and lifestyles may differ; therefore, it may not be appropriate to apply universal interventions to people in a state of continuous SI. However, we did not detect any clusters in which only participants with a particular transition pattern of SI were accounted for, and clusters with many improved-SI or worsened-SI groups were not extracted. Therefore, the transition pattern of SI may not have contributed much to the clustering of participants in this study; thus, these results should be interpreted with caution.

As this study and other previous studies have shown, SI during the COVID-19 pandemic has been severe; therefore, it should be urgently addressed to protect people's mental health. However, research on intervening in SI or loneliness during the pandemic has not been sufficiently conducted [40]. This study showed various possible factors that contribute to SI, and the causal relationship between SI and these factors may not be uniform. Thus, intervention methods will differ depending on each person's SI experience background. This study demonstrates the necessity of careful assessment of the psychological, social, and behavioral characteristics associated with SI to evaluate the mechanisms of SI in each individual and intervene appropriately. Therefore, this study’s results will be beneficial for developing intervention methods that fit the characteristics of individuals for those who are socially isolated during a pandemic.

### Limitations

This study had several limitations. First, we did not assess the quality of relationships with relatives and friends. Even if the network size is small, mental health may be good if the quality of the relationships is sufficient. Second, we did not exclude people who did not stay in a mild lockdown for any reason (eg, work) and people affected by COVID-19, and we could not adjust for their effect on the results of the study. In the future, it would be useful to investigate whether the participants were in an environment affected by the mild lockdown or COVID-19. Third, we collected the data for this study through an online survey and could not conduct random sampling. Thus, we cannot guarantee the sample’s representativeness, which could not be matched to the proportions of each age group and gender group in each region. Additionally, populations registered with online survey companies may be more willing to participate in surveys than nonregistered populations. They may have social networks to obtain information about such survey cooperation. There may be more people with severe SI in the nonenrolled population who could have different characteristics and need additional support from the findings of this study. Fourth, the significant differences between people who responded in phases 1 and 2 and people who responded only in phase 1 were indicated in some sociodemographic characteristics and psychological variables. Fifth, items on the treatment of psychological problems and physical diseases asked only about their presence or absence, and the definition of these problems was left to the participants. These differences may have caused a selection bias. Therefore, it was up to the participants to decide whether psychosomatic disorders, for example, were included in either category.

### Conclusion

We longitudinally investigated the transition of SI and its related factors by surveying during the mild lockdown under Japan’s 2 declared states of emergency. When the second declaration occurred, more than half of the population was socially isolated. Moreover, many people became socially isolated between the first and second declarations, particularly low-income, unmarried, or childless individuals. Among individuals with persistent SI, there were 2 groups: those whose mental health was deteriorating and those whose mental health was not deteriorating, with the former having more problems with relationships and interactions. This study’s results emphasized variables that should be evaluated explicitly in interventions for SI during a pandemic and may help develop more effective intervention methods tailored to each person’s characteristics.

## Appendix

Multimedia Appendix 1 Items about lifestyle, coping behavior, and stressors related to mild lockdown.

Multimedia Appendix 2 Comparisons of sex ratio, age, and psychological indexes between individuals who participated both in phase 1 and 2 and individuals who participated only in phase 1.

Multimedia Appendix 3 Characteristics of psychosocial variables in each cluster.

## Abbreviations

K6: Kessler Psychological Distress Scale-6
LSNS-6: Lubben Social Network Scale (shortened version)
PHQ-9: Patient Health Questionnaire-9
SI: social isolation
UCLA-LS3: University of California, Los Angeles (UCLA) Loneliness Scale, Version 3
