# Correction: The Transition of Social Isolation and Related Psychological Factors in 2 Mild Lockdown Periods During the COVID-19 Pandemic in Japan: Longitudinal Survey Study

**DOI:** 10.2196/39498

**Published:** 2022-05-16

**Authors:** Nagisa Sugaya, Tetsuya Yamamoto, Naho Suzuki, Chigusa Uchiumi

**Affiliations:** 1 Unit of Public Health and Preventive Medicine School of Medicine Yokohama City University Yokohama Japan; 2 Graduate School of Technology, Industrial and Social Sciences Tokushima University Tokushima Japan; 3 Graduate School of Sciences and Technology for Innovation Tokushima University Tokushima Japan

In “The Transition of Social Isolation and Related Psychological Factors in 2 Mild Lockdown Periods During the COVID-19 Pandemic in Japan: Longitudinal Survey Study” (JMIR Public Health Surveill 2022;8(3):e32694) the authors noted one error.

In Table 2 of the originally published article, the standard deviations of the PHQ-9 in phases 1 and 2 in the 'Persistent-SI' group were missed. The table was originally published as shown in Multimedia Appendix 1.

The 2 SD values were 5.93 and 6.07, respectively, and the corrected Table 2 is as follows:

**Table 2:** Differences and interactions between phases^a^ and transition of SI^b^ on different scales.

	Phase	Mean score (SD)					Effect of phase				Effect of group				Interaction		
		No SI	Improved SI	Worsened SI	Persistent SI	*F* (*df*)		*P* value	*η_G^2^_^c^*	*F* (*df*)		*P* value	*η_G^2^_*	*F* (*df*)		*P* value	*η_G^2^_*
**LSNS-6^d^**																	
	1	16.84 (3.71)	7.89 (2.77)	14.58 (2.73)	5.50 (3.31)	15.18 (1, 7889)		<.001	0.000	8071.80 (3, 7889)		<.001	0.640	2046.36 (3, 7889)		<.001	0.066
	2	16.46 (3.55)	14.56 (2.83)	7.81 (2.93)	5.21 (3.28)	N/A^e^		N/A	N/A	N/A		N/A	N/A	N/A		N/A	N/A
**UCLA-LS3^f^**																	
	1	19.56 (4.67)	23.07 (4.61)	22.26 (4.55)	26.41 (5.20)	10.51 (1, 7889)		.001	0.000	1096.28 (3, 7889)		<.001	0.259	38.59 (3, 7889)		<.001	0.002
	2	19.87 (4.86)	22.13 (4.76)	23.38 (4.59)	26.62 (5.33)	N/A		N/A	N/A	N/A		N/A	N/A	N/A		N/A	N/A
**K6^g^**																	
	1	3.99 (4.38)	5.23 (5.37)	4.96 (4.88)	6.12 (5.77)	536.70 (1, 7889)		<.001	0.013	103.10 (3, 7889)		<.001	0.030	2.64 (3, 7889)		.048	0.000
	2	2.63 (4.01)	3.37 (4.87)	3.68 (4.82)	4.71 (5.63)	N/A		N/A	N/A	N/A		N/A	N/A	N/A		N/A	N/A
**PHQ-9^h^**																	
	1	3.05 (4.06)	4.41 (5.39)	4.19 (5.05)	5.53 (5.93)	168.90 (1, 7889)		<.001	0.004	126.46 (3, 7889)		<.001	0.038	2.32 (3, 7889)		.073	0.000
	2	2.4 (3.89)	3.27 (5.19)	3.49 (4.88)	4.77 (6.07)	N/A		N/A	N/A	N/A		N/A	N/A	N/A		N/A	N/A

^a^Phase 1: between May 11 and 12, 2020, in the final phase of the first state of emergency; phase 2: between February 24 and 28, 2021, in the final phase of the second state of emergency.

^b^SI: social isolation.

^c^*η_G_^2^*: 0.010, small; 0.060, medium; 0.140, large.

^d^LSNS-6: Lubben Social Network Scale (shortened version).

^e^N/A: not applicable.

^f^UCLA-LS3: University of California, Los Angeles (UCLA) Loneliness Scale, Version 3.

^g^K6: Kessler Psychological Distress Scale-6.

^h^PHQ-9: Patient Health Questionnaire-9.

The correction will appear in the online version of the paper on the JMIR Publications website on May 16, 2022, together with the publication of this correction notice. Because this was made after submission to PubMed, PubMed Central, and other full-text repositories, the corrected article has also been resubmitted to those repositories.

## Appendix

Multimedia Appendix 1 Original published version of “Table 2. Differences and interactions between phases^a^ and transition of SI^b^ on different scales.”

