# Supplementary material for: The Transition of Social Isolation and Related Psychological Factors in 2 Mild Lockdown Periods During the COVID-19 Pandemic in Japan: Longitudinal Survey Study
Source: JMIR Public Health Surveill. 2022 Mar 8;8(3):e32694. doi: 10.2196/32694 (PMC8906839; doi:10.2196/32694)
Supplement: Multimedia Appendix 2 [file publichealth_v8i3e32694_app2.docx]

Appendix 2. Comparisons of sex ratio, age, and psychological indexes between individuals who participated both in phase 1 and 2 and individuals who participated only in phase 1

|  | Participated in phase 1 & 2 (n=7893) | Participated only in phase 1 (n=3440) | *χ*^2^ or Difference (95%CI) | *p* | *φ* or Cohen's *d* |
| --- | --- | --- | --- | --- | --- |
| Women, n (%) | 3692 (46.8%) | 2250 (65.4%) | 333.45 | <.001 | 0.172 |
| Age, mean (SD) | 49.56 (13.71) | 38.66 (13.55) | -10.90 (-11.45, -10.36) | <.001 | 0.8 |
| LSNS-6, mean (SD) | 10.14 (6.13) | 11.53 (6.15) | 1.40 (1.15, 1.64) | <.001 | 0.227 |
| UCLA-LS3, mean (SD) | 23.59 (5.74) | 23.17 (5.59) | 0.41 (-0.64, -0.19) | <.001 | 0.073 |
| K6, mean (SD) | 5.27 (5.34) | 6.28 (5.58) | 1.01 (0.79, 1.23) | <.001 | 0.184 |
| PHQ-9, mean (SD) | 4.54 (5.40) | 5.74 (5.75) | 1.20 (0.97, 1.43) | <.001 | 0.215 |

LSNS-6, Lubben Social Network Scale (abbreviated version); UCLA-LS3, UCLA Loneliness Scale (version 3); K6, Kessler Psychological Distress Scale-6; PHQ-9, Patient Health Questionnaire-9

*φ*: 0.100~ small; 0.300~ medium; 0.600~ large

Cohen’s *d*: 0.200~ small; 0.500~ medium; 0.800~ large
