# Supplementary material for: The Transition of Social Isolation and Related Psychological Factors in 2 Mild Lockdown Periods During the COVID-19 Pandemic in Japan: Longitudinal Survey Study
Source: JMIR Public Health Surveill. 2022 Mar 8;8(3):e32694. doi: 10.2196/32694 (PMC8906839; doi:10.2196/32694)
Supplement: Multimedia Appendix 3 [file publichealth_v8i3e32694_app3.docx]

Appendix 3 Characteristics of psychosocial variables in each cluster


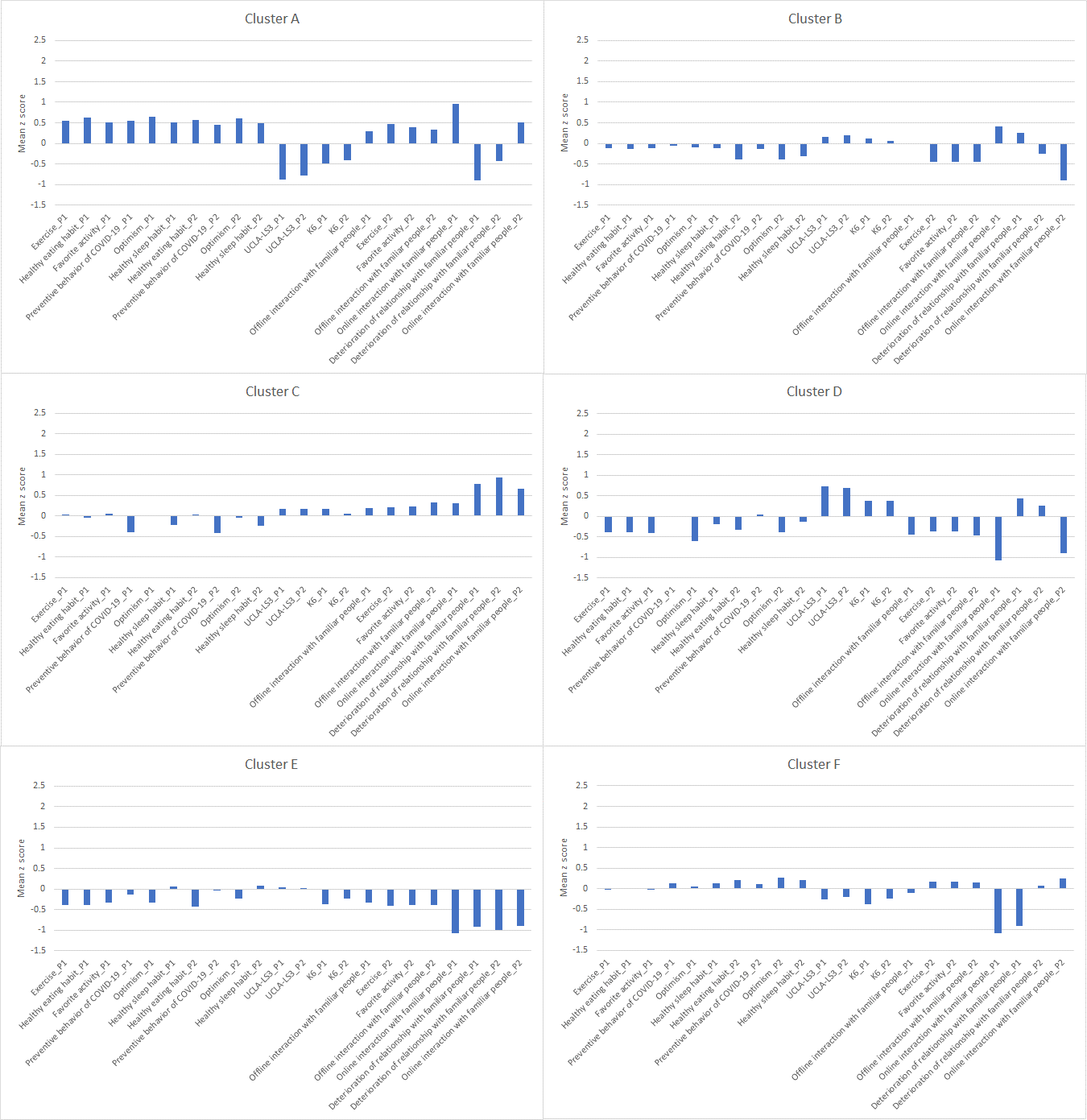


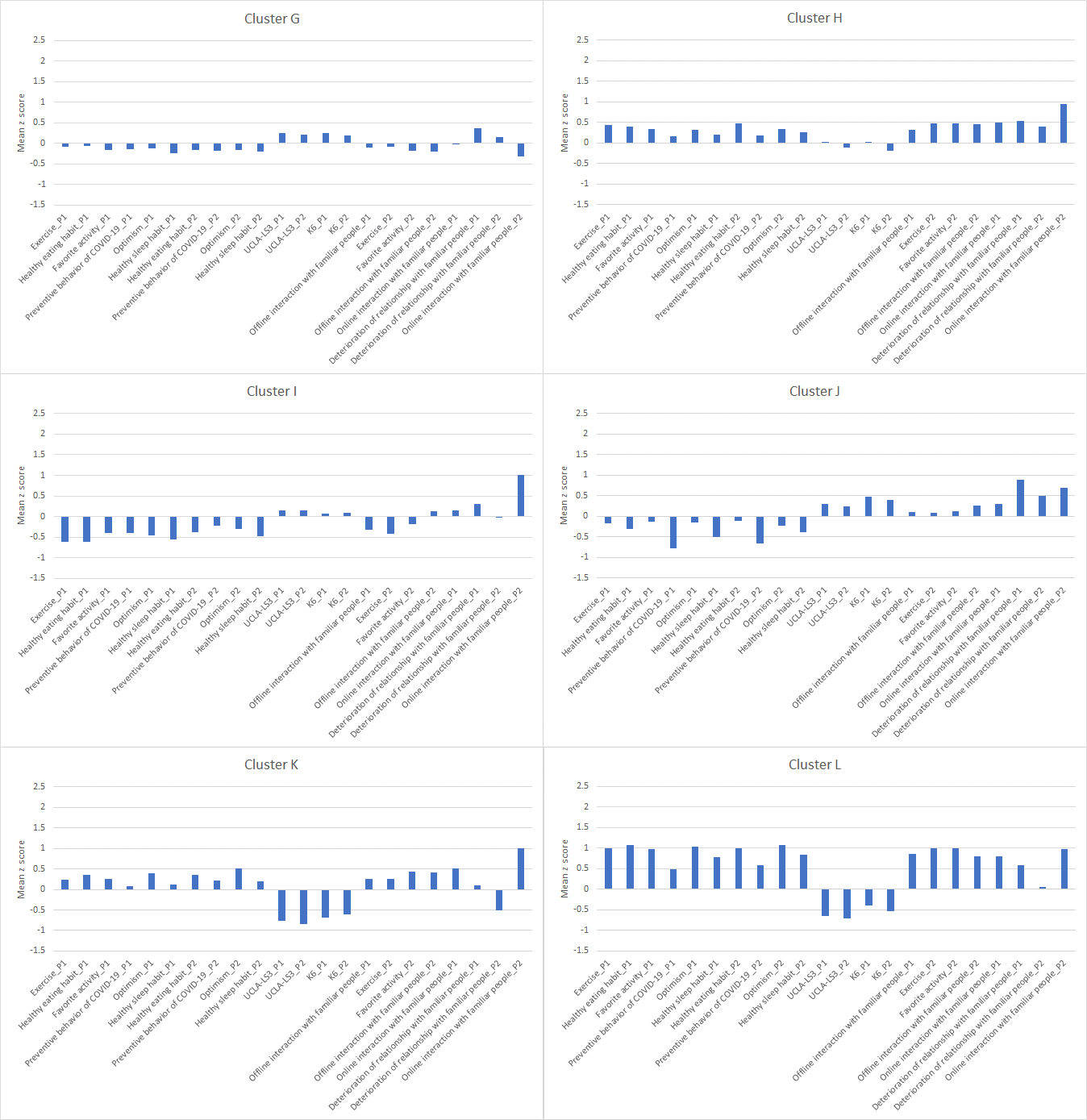


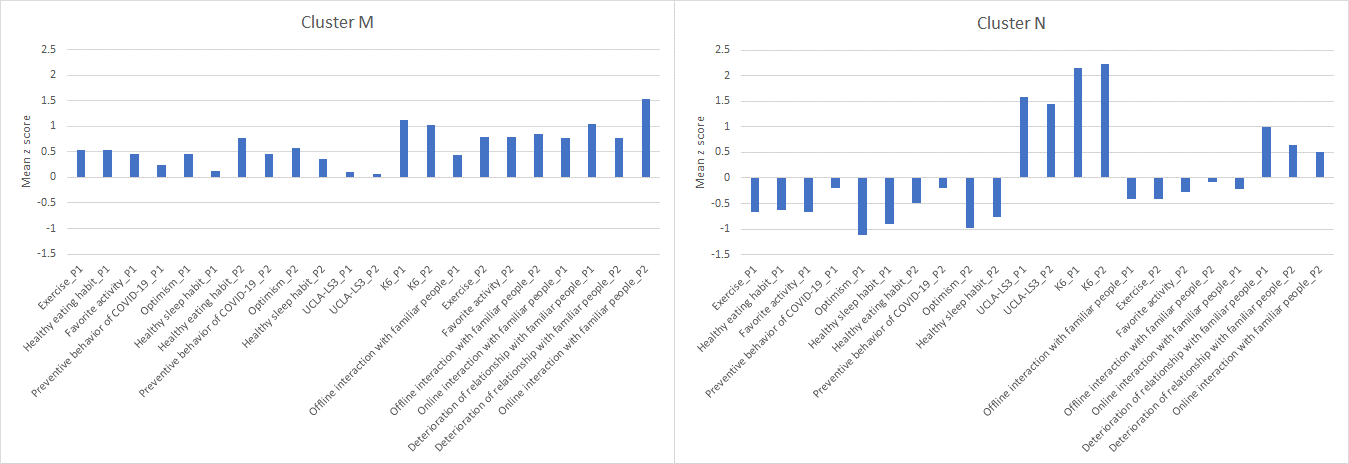


The red dashed lines indicate each cluster of variables.

UCLA-LS3, UCLA Loneliness Scale (version 3); K6, Kessler Psychological Distress Scale-6; COVID-19, coronavirus disease 2019; P1: Phase 1; P2: Phase 2
