# Supplementary material for: Correction: The Transition of Social Isolation and Related Psychological Factors in 2 Mild Lockdown Periods During the COVID-19 Pandemic in Japan: Longitudinal Survey Study
Source: JMIR Public Health Surveill. 2022 May 16;8(5):e39498. doi: 10.2196/39498 (PMC9152719; doi:10.2196/39498)
Supplement: Multimedia Appendix 1 [file publichealth_v8i5e39498_app1.docx]

**Multimedia Appendix 1:** Original published version of “Table 2. Differences and interactions between phases^a^ and transition of SI^b^ on different scales.”

| Phase | | Mean score (SD) | | | | Effect of phase | | | Effect of group | | | Interaction | | |
| --- | --- | --- | --- | --- | --- | --- | --- | --- | --- | --- | --- | --- | --- | --- |
|  |  | No SI | Improved SI | Worsened SI | Persistent SI | *F* (*df*) | *P* value | η_G_^2 c^ | *F* (*df*) | *P* value | η_G_^2^ | *F* (*df*) | *P* value | η_G_^2^ |
|  |  |  |  |  |  |  |  |  |  |  |  |  |  |  |
| **LSNS-6^d^** | | | | | | | | | | | | | | |
|  | 1 | 16.84 (3.71) | 7.89 (2.77) | 14.58 (2.73) | 5.50 (3.31) | 15.18 (1, 7889) | <.001 | 0.000 | 8071.80 (3, 7889) | <.001 | 0.640 | 2046.36 (3, 7889) | <.001 | 0.066 |
|  | 2 | 16.46 (3.55) | 14.56 (2.83) | 7.81 (2.93) | 5.21 (3.28) | N/A^e^ | N/A | N/A | N/A | N/A | N/A | N/A | N/A | N/A |
| **UCLA-LS3^f^** | | | | | | | | | | | | | | |
|  | 1 | 19.56 (4.67) | 23.07 (4.61) | 22.26 (4.55) | 26.41 (5.20) | 10.51 (1, 7889) | .001 | 0.000 | 1096.28 (3, 7889) | <.001 | 0.259 | 38.59 (3, 7889) | <.001 | 0.002 |
|  | 2 | 19.87 (4.86) | 22.13 (4.76) | 23.38 (4.59) | 26.62 (5.33) | N/A | N/A | N/A | N/A | N/A | N/A | N/A | N/A | N/A |
| **K6^g^** | | | | | | | | | | | | | | |
|  | 1 | 3.99 (4.38) | 5.23 (5.37) | 4.96 (4.88) | 6.12 (5.77) | 536.70 (1, 7889) | <.001 | 0.013 | 103.10 (3, 7889) | <.001 | 0.030 | 2.64 (3, 7889) | .048 | 0.000 |
|  | 2 | 2.63 (4.01) | 3.37 (4.87) | 3.68 (4.82) | 4.71 (5.63) | N/A | N/A | N/A | N/A | N/A | N/A | N/A | N/A | N/A |
| **PHQ-9^h^** | | | | | | | | | | | | | | |
|  | 1 | 3.05 (4.06) | 4.41 (5.39) | 4.19 (5.05) | 5.53 (.) | 168.90 (1, 7889) | <.001 | 0.004 | 126.46 (3, 7889) | <.001 | 0.038 | 2.32 (3, 7889) | .073 | 0.000 |
|  | 2 | 2.4 (3.89) | 3.27 (5.19) | 3.49 (4.88) | 4.77 | N/A | N/A | N/A | N/A | N/A | N/A | N/A | N/A | N/A |

^a^Phase 1: between May 11 and 12, 2020, in the final phase of the first state of emergency; phase 2: between February 24 and 28, 2021, in the final phase of the second state of emergency.

^b^SI: social isolation.

^c^ηG 2 : 0.010, small; 0.060, medium; 0.140, large.

^d^LSNS-6: Lubben Social Network Scale (shortened version).

^e^N/A: not applicable.

^f^UCLA-LS3: University of California, Los Angeles (UCLA) Loneliness Scale, Version 3.

^g^K6: Kessler Psychological Distress Scale-6.

^h^PHQ-9: Patient Health Questionnaire-9.
